# Functional and Structural Insights into the Human PPARα/δ/γ Targeting Preferences of Anti-NASH Investigational Drugs, Lanifibranor, Seladelpar, and Elafibranor

**DOI:** 10.3390/antiox12081523

**Published:** 2023-07-29

**Authors:** Shotaro Kamata, Akihiro Honda, Ryo Ishikawa, Makoto Akahane, Ayane Fujita, Chihiro Kaneko, Saeka Miyawaki, Yuki Habu, Yui Shiiyama, Kie Uchii, Yui Machida, Takuji Oyama, Isao Ishii

**Affiliations:** 1 Department of Health Chemistry, Showa Pharmaceutical University, Machida 194-8543, Tokyo, Japan; 2 Faculty of Life and Environmental Sciences, University of Yamanashi, Kofu 400-8510, Yamanashi, Japan

**Keywords:** NAFLD, NASH, PPAR subtypes, dual/pan agonist, ligand-binding domain, X-ray crystallography

## Abstract

No therapeutic drugs are currently available for nonalcoholic steatohepatitis (NASH) that progresses from nonalcoholic fatty liver via oxidative stress-involved pathways. Three cognate peroxisome proliferator-activated receptor (PPAR) subtypes (PPARα/δ/γ) are considered as attractive targets. Although lanifibranor (PPARα/δ/γ pan agonist) and saroglitazar (PPARα/γ dual agonist) are currently under investigation in clinical trials for NASH, the development of seladelpar (PPARδ-selective agonist), elafibranor (PPARα/δ dual agonist), and many other dual/pan agonists has been discontinued due to serious side effects or little/no efficacies. This study aimed to obtain functional and structural insights into the potency, efficacy, and selectivity against PPARα/δ/γ of three current and past anti-NASH investigational drugs: lanifibranor, seladelpar, and elafibranor. Ligand activities were evaluated by three assays to detect different facets of the PPAR activation: transactivation assay, coactivator recruitment assay, and thermal stability assay. Seven high-resolution cocrystal structures (namely, those of the PPARα/δ/γ-ligand-binding domain (LBD)–lanifibranor, PPARα/δ/γ-LBD–seladelpar, and PPARα-LBD–elafibranor) were obtained through X-ray diffraction analyses, six of which represent the first deposit in the Protein Data Bank. Lanifibranor and seladelpar were found to bind to different regions of the PPARα/δ/γ-ligand-binding pockets and activated all PPAR subtypes with different potencies and efficacies in the three assays. In contrast, elafibranor induced transactivation and coactivator recruitment (not thermal stability) of all PPAR subtypes, but the PPARδ/γ-LBD–elafibranor cocrystals were not obtained. These results illustrate the highly variable PPARα/δ/γ activation profiles and binding modes of these PPAR ligands that define their pharmacological actions.

## 1. Introduction

There is serious concern regarding the medical/economic burden of the treatment of the globally expanding number of patients with nonalcoholic fatty liver disease (NAFLD)/nonalcoholic steatohepatitis (NASH). NAFLD is defined by the evidence of hepatic steatosis (either through imaging or histology) and the absence of secondary causes of significant alcohol consumption, long-term use of a steatogenic medication, or monogenic hereditary disorders [1]. The overall prevalence of nonalcoholic fatty liver (NAFL) worldwide is estimated to be 32.4% [2], while an estimated 10–25% of NAFL patients progress to the development of NASH (a condition characterized by ≥5% hepatic steatosis and inflammation with hepatic injury (e.g., ballooning) in the presence or absence of fibrosis [1]) in which oxidative stress plays a pivotal role by stimulating Kupffer cells, hepatic stellate cells, and hepatocytes [3]. NASH can further progress to cirrhosis, end-stage liver disease, or hepatocellular carcinoma [4], and NAFLD is the leading cause of liver-related morbidity and mortality. NASH is the major cause of liver transplantation and, currently, there are no approved non-symptomatic therapies for NASH by the Food and Drug Administration (FDA) or the European Medicines Agency [5]. NASH is a multifaceted condition with variable coexisting metabolic complications such as obesity and type 2 diabetes, thereby further complicating its treatment [4]. Its therapeutic targets can be divided into four major categories: (i) metabolic targets, (ii) targets related to inflammation or cell injury, (iii) liver–gut axis targets, and (iv) targets related to fibrosis [5,6]. In this respect, the peroxisome proliferator-activated receptors (PPARs) are attractive therapeutic targets that could simultaneously improve steatosis, ballooning, inflammation, and fibrosis [6,7].

PPARs belong to the nuclear receptor superfamily and the ligand-activated transcription factors; they exist in three subtypes in mammals (namely, PPARα, PPARβ/δ, and PPARγ) with considerable amino acid identity (54–71% in humans). The synthetic PPARα agonists known as “fibrates” have been widely used for the treatment of hypertriglyceridemia, while the synthetic PPARγ agonists known as “thiazolidinediones (glitazones)” are anti-diabetic drugs. In the guidelines of the American Association for the Study of Liver Diseases, pioglitazone has been proposed for the treatment of biopsy-proven NASH. The PPAR pan agonist lanifibranor (IVA-337) and the PPARα/γ dual agonist saroglitazar are currently in clinical trials for NASH (phase 3 in NCT04849728 and 2b in NCT05011305, respectively). However, the development of most glitazars (PPARα/γ dual agonists), including muraglitazar, tesaglitazar, and aleglitazar, has been abandoned due to serious safety concerns [8]. The use of the PPARδ-selective agonist seladelpar (MBX-8025) for the treatment of NASH has been once discontinued at phase 2b [9], while that of the PPARα/δ dual agonist elafibranor against NASH has been discontinued due to its non-significant benefits [10]. Therefore, the risks and the benefits of each PPAR-targeting drug should be carefully discussed based on detailed analyses at molecular levels.

This study was designed so as to provide functional and structural insights into the potency, efficacy, and selectivity against PPARα/δ/γ of lanifibranor, seladelpar, and elafibranor. We have found that all three agents can activate all PPAR subtypes with highly different preferences in the three different PPAR activation assays undertaken herein. Furthermore, we have characterized the high-resolution structures of the PPARα/δ/γ-ligand-binding domain (LBD)–lanifibranor, the PPARα/δ/γ-LBD–seladelpar, and the PPARα-LBD–elafibranor cocrystals through X-ray diffraction analyses.

## 2. Materials and Methods

### 2.1. PPAR Activation Assay 1: Transactivation Assay

In order to evaluate the PPARα/δ/γ-mediated transcriptional activation, pSG5-GAL–human PPARα/δ/γ chimera expression plasmids, a MH100(UAS)×4-tk-Luc reporter plasmid, and a pRL-CMV *Renilla* luciferase control plasmid under the control of a cytomegalovirus promoter were cotransfected into COS-7 cells (No. RCB0539; Riken BRC Cell Bank, Tsukuba, Ibaraki, Japan) that were maintained in Dulbecco’s modified Eagle’s medium (DMEM) supplemented with 10% fetal bovine serum (FBS) and antibiotics, at 37 °C, in a 5% CO_2_/95% air incubator. The pSG5-GAL–hPPARα/δ/γ plasmids express fusion proteins comprising the yeast transcription factor GAL4 DNA-binding domain and each of the human PPARα/δ/γ-LBDs [11,12]. The MH100(UAS)×4-tk-Luc plasmid contains four copies of the MH100 GAL4 binding site and the *Firefly* luciferase gene [13]. The cells were transfected with those plasmids and were treated with various PPAR ligands, and both *Firefly* and *Renilla* luciferase activities were measured using the Dual-Glo Luciferase Assay System (Promega, Madison, WI, USA) as described previously [14]. The transactivation activities were expressed as percentages of the maximal *Firefly* luciferase responses induced by potent/specific PPARα/δ/γ agonists: GW7647 (1 µM) for PPARα, GW501516 (0.02 µM) for PPARδ, and GW1929 (1 µM) for PPARγ [14], after normalization with the *Renilla* luciferase responses. GW7647, GW501516, and pioglitazone were purchased from the Cayman Chemical Company (Ann Arbor, MI, USA). Elafibranor, lanifibranor, seladelpar, and saroglitazar were purchased from ChemScene (Monmouth Junction, NJ, USA). GW1929 was purchased from Sigma-Aldrich (St. Louis, MO, USA).

### 2.2. Recombinant PPARα/δ/γ-LBD Expression and Purification

Human PPARα-LBD (amino acids 200–468), PPARδ-LBD (amino acids 170–441), and PPARγ-LBD (amino acids 203–477 in isoform 1) were expressed as amino-terminal His-tagged proteins by the pET28a vector (Novagen, Madison, WI, USA) in Rosetta (DE3) pLysS competent cells (Novagen) and were subsequently purified using three-step chromatography, as previously described in detail [14,15,16,17].

### 2.3. PPAR Activation Assay 2: PGC1α/SRC1 Coactivator Recruitment Assay

The activation status of each PPARα/δ/γ subtype was also determined by a time-resolved fluorescence resonance energy transfer (TR-FRET) assay that detects physical interactions between His-tagged hPPARα/δ/γ-LBD proteins and a biotin-labeled PPARγ coactivator 1α (PGC1α) coactivator peptide (biotin-EAEEPSLLKKLLLAPANTQ (amino acids 137–155) synthesized by GenScript) or a steroid receptor coactivator 1 (SRC1) peptide (biotin-CPSSHSSLTERHKILHRLLQEGSPS (amino acids 676–700) from GenScript) using the LANCE Ultra TR-FRET assay (PerkinElmer, Waltham, MA, USA) [14,15,17]. A 9.5 µL aliquot of PPARα/δ/γ-LBD (400 nM in buffer A consisting of 10 mM HEPES-NaOH (pH 7.4), 150 mM NaCl, 0.005% Tween 20, and 0.1% fatty acid-free bovine serum albumin (BSA) for PPARα/γ-LBD; 400 nM in buffer B consisting of 50 mM HEPES-NaOH (pH 7.4), 50 mM KCl, 1 mM EDTA, 0.5 mM dithiothreitol, and 0.1% fatty acid-free BSA for PPARδ-LBD), 0.5 µL of a 100× ligand solution (in DMSO), and 5 µL of biotin-PGC1α (4 µM) or biotin-SRC1 peptide (8 µM) were mixed in a well of Corning 384-well low-volume, white, round-bottom, polystyrene non-binding surface microplate. Subsequently, 5 µL of 4 nM Eu-W1024-labeled anti-6×His antibody/80 nM ULight-Streptavidin (PerkinElmer) was added to each well, and the microplate was incubated in the dark for 2 h, at room temperature. FRET signals were detected at one excitation filter (340/12) and at two emission filters (615/12 and 665/12) using a Varioskan Flash double monochromator microplate reader (Thermo Fisher Scientific, Waltham, MA, USA). The parameters for the measurements at 615 and 665 nm were an integration time of 200 µs and a delay time of 100 µs. The 665 nm emissions were due to ULight-FRET, while the 615 nm emissions were due to Eu-W1024. The 665/615 ratio was calculated and normalized to the negative control reaction using 1% DMSO. The nonlinear fitting and the calculation of the EC_50_ were performed using the Prism 9 software (GraphPad, San Diego, CA, USA).

### 2.4. PPAR Activation Assay 3: Thermal Stability Assay Using Circular Dichroism Spectroscopy

PPARα/δ/γ-LBD proteins (10 µM) were incubated with different concentrations of ligands in buffer C consisting of 20 mM Tris-HCl (pH 8.0), 150 mM NaCl, 1 mM Tris 2-carboxyethylphosphine (TCEP)-HCl, and 10% glycerol. The circular dichroism (CD) spectra were monitored within a 200–260 nm range at increasing temperatures from 30 °C to 70 °C (2 °C/min) using a J-1500 spectropolarimeter equipped with a PTC-510 thermal controller (JASCO, Tokyo, Japan). The spectra of all PPARs displayed local minima at 208 and 222 nm, which is a typical feature of α-helical proteins [18]. The thermal stability of the PPARs was investigated by continuously monitoring the ellipticity changes at 222 nm during the thermal denaturation [14,15], and a single-site sigmoidal dose–response curve fitting program (Prism 9) was used in order to obtain the melting temperature (*T*_m_) that corresponds to the midpoint of the denaturation process. The ligand-induced increases in the *T*_m_ values were defined as ∆*T*_m_.

### 2.5. Cocrystallization of PPARα/δ/γ-LBD with Lanifibranor, Seladelpar, or Elafibranor

#### 2.5.1. PPARα Cocrystals

We have previously obtained the PPARα-LBD–clofibric acid (a low affinity PPARα ligand with an EC_50_ value of 574 µM in the PGC1α recruitment activity) [15]–SRC1 (LTERHKILHRLLQEG; (amino acids 683–697) from GenScript, Piscataway, NJ, USA) cocrystal by cocrystallization with delipidized PPARα [16], and have revealed its high-resolution (2.09-Å) structure (PDB ID: 7BPY) [15]. The PPARα-LBD–clofibric acid–SRC1 cocrystals were soaked in a reservoir solution (0.1 M HEPES (pH 7.5)/20% PEG3350) containing 2 mM of lanifibranor or elafibranor (final 10% DMSO) at 4 °C, for three days, in order to obtain cocrystals with each ligand. The cocrystallization with seladelpar was performed in hanging-drop mixtures of 0.5 µL PPARα-LBD (20 mg/mL in buffer C), 0.5 µL ligand (2 mM in buffer C), and 1 µL reservoir solution (0.1 M Tris (pH 8.5)/25% PEG3350), at 4 °C, for several weeks.

#### 2.5.2. PPARδ Cocrystals

The cocrystallization of PPARδ-LBD–lanifibranor/seladelpar was performed in hanging-drop mixtures of 0.5 µL of PPARδ-LBD (10 mg/mL in buffer C), 0.1 µL of 10 mM ligand, 0.3 µL of buffer D (20 mM Tris-HCl (pH 8.0), 500 mM ammonium acetate, 1 mM TCEP-HCl, and 10% glycerol), 0.1 µL of 5% *n*-octyl-β-d-glucoside, and 1 µL reservoir solution (50 mM Bis-Tris propane (pH 7.5), 14% PEG8000, 0.2 M KCl, 6% propanediol, 1 mM EDTA, 1 mM CaCl_2_ for PPARδ-LBD–lanifibranor; 50 mM Bis-Tris propane (pH 8.0), 14% PEG8000, 0.1 M KSCN, 6% propanediol, 1 mM EDTA, 1 mM CaCl_2_ for PPARδ-LBD–seladelpar), at 20 °C, for several weeks.

#### 2.5.3. PPARγ Cocrystals

The cocrystallization of PPARγ-LBD–lanifibranor/seladelpar–SRC1 was performed in hanging-drop mixtures of 0.5 µL PPARγ-LBD (20 mg/mL in buffer C), 0.5 µL ligand (2 mM in buffer C), and 1 µL reservoir solution (0.1 M HEPES-NaOH (pH 7.5)/1.0 M trisodium citrate dihydrate for PPARγ-LBD–lanifibranor–SRC1; 0.1 M Tris (pH 8.0)/1.1 M trisodium citrate dihydrate for PPARγ-LBD–seladelpar–SRC1), at 20 °C, for several weeks.

All obtained cocrystals were briefly soaked in a cryoprotection buffer (each reservoir solution plus 20% glycerol for PPARα/δ-LBD crystals and 30% glycerol for PPARγ-LBD crystals). Subsequently, these were flash-cooled in a stream of liquid nitrogen until the X-ray diffraction analysis was conducted.

### 2.6. X-ray Diffraction: Data Collection and Model Refinement

Datasets were collected by a BL-5A or a BL-17A beamline at the Photon Factory (Tsukuba, Ibaraki, Japan) using synchrotron radiation of 1.0 Å. Diffraction data were collected at a 0.1° oscillation per frame, and a total of 1800 frames (180°) were recorded for a 1.0-Å X-ray crystallography [14,15,17]. Data processing and scaling were carried out using the XDS X-ray detector software (version February 5, 2021) [19] and AIMLESS (version 0.5.21) [20], respectively. Resolution cutoff values (*R*_merge_ < 0.5, *R*_pim_ < 0.3, and completeness > 0.9) were set by the highest resolution shell [14,15,17]. All structures were determined by using the molecular replacement in PHASER (version 2.7.16) [21] with Protein Data Bank (PDB) IDs: 3SP6 for PPARα-LBD, 2ZNQ or 7WGL for PPARδ-LBD, and 1WM0 or 7WGO for all PPARγ-LBDs as the search model. Refinement was performed using the iterative cycles of the model adjustment in two programs: COOT (version 0.8.2) [22] and PHENIX (version 1.11.1-2575-000) [23]. The structures were constructed using the PyMOL program (version 2.5.0). All collection data and refinement statistics are summarized in Supplementary Materials Table S1.

### 2.7. Evaluation of Molecular Interactions between PPAR-LBD Amino Acids and Ligands

Based on those X-ray cocrystal structures, proximity distances between each amino acid in PPARα/δ/γ-LBD and the three ligands were measured using PyMOL. All PPARα/δ/γ-LBD amino acids that have 4.5 Å or less proximity distances from the ligands were listed in Table S2. All molecular interactions between those amino acids and the ligands were evaluated with the MolDock scores using Ligand Energy Inspector programs in Molegro Virtual Docker (MVD) software (version 6.0; CLC bio, Aarhus, Denmark) (Table S2). The scoring function of MolDock is based on an extended piecewise linear potential including new hydrogen bonding and electrostatic terms.

## 3. Results

### 3.1. Transactivation of Gene Expression via PPARα/δ/γ-LBD

**Figure 1:**
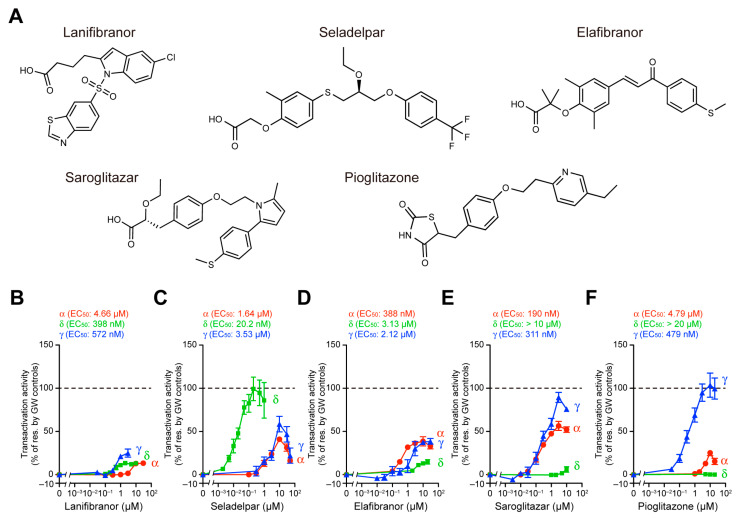
LBD-mediated human PPARα/δ/γ transactivation by PPAR ligands. (**A**) Chemical structures of the five PPAR ligands used in this study. (**B**–**F**) LBD-mediated PPARα/δ/γ transactivation was induced in COS-7 cells by PPARα-selective GW7647, PPARδ-selective GW501516, and PPARγ-selective GW1929 in a concentration-dependent manner, and their maximal responses (at 1, 0.02, and 1 µM, respectively) were considered as 100% (dashed lines) of the responses. Concentration-dependent relative transactivation activities by lanifibranor (**B**), seladelpar (**C**), elafibranor (**D**), saroglitazar (**E**), and pioglitazone (**F**) are shown as percent responses with calculated EC_50_ values. Data are presented as the mean ± standard error (SE) of three independent experiments with duplicate samples.

For the undertaking of the comparison of the potency, efficacy, and selectivity of the five PPAR ligands (Figure 1A) against the PPAR subtypes, we evaluated their PPARα/δ/γ activation using three different methods: a cell-based transactivation assay, a coactivator recruitment assay, and a thermal stability assay, as we previously performed on three fibrates (namely, bezafibrate, fenofibric acid, and pemafibrate) [14]. The cell-based transactivation assay utilizing the Gal4–PPAR-LBD system is the most widely used method for the determination of PPAR activation by certain ligands, although their responses depend on the used cell types in which several coactivators with altered functions are differentially expressed [24,25,26]. The maximal effects of the PPAR-selective full agonists (1 µM GW7647 for PPARα, 0.02 µM GW501516 for PPARδ, and 1 µM GW1929 for PPARγ) [14] in COS-7 cells were considered as the 100% transactivation responses. The “so-called” PPAR pan agonist lanifibranor [27] indeed activated all PPAR subtypes with EC_50_ values of 398 nM (for δ) < 572 nM (for γ) < 4.66 µM (for α), and equivalent efficacies (13.3% of the maximal responses for α/δ and 24.9% for γ) (Figure 1B). The PPARδ-selective agonist seladelpar [28] activated PPARδ with the highest potency (EC_50_: 20.2 nM) and efficacy (99.3%) compared to PPARα (1.64 µM and 41.0%) and PPARγ (3.53 µM and 58.5%) (Figure 1C). Unexpectedly, the PPARα/δ dual agonist elafibranor [29] activated all PPAR subtypes with potencies (EC_50_) of 388 nM (for α) < 2.12 µM (for γ) < 3.13 µM (for δ) and efficacies of 38.5% (for α/γ) > 14.8% (for δ), thereby acting like a PPARα/γ dual agonist (Figure 1D). In contrast, the genuine PPARα/γ dual agonist saroglitazar [17] activated the PPARα/γ at higher potencies (190 nM/311 nM vs. > 10 µM in δ) and efficacies (56.4%/89.4% vs. 6.0% in δ) (Figure 1E). The PPARγ-selective full agonist pioglitazone [30] indeed activated PPARγ with the highest potency (EC_50_: 479 nM) and efficacy (104%) compared to PPARα (4.79 µM and 25.2%) and PPARδ (>20 µM and 0.518%) (Figure 1F). These findings reveal that four of the five assessed PPAR ligands (i.e., except for elafibranor) act much as expected.

### 3.2. PGC1α/SRC1 Coactivator Recruitment via PPARα/δ/γ-LBD

**Figure 2:**
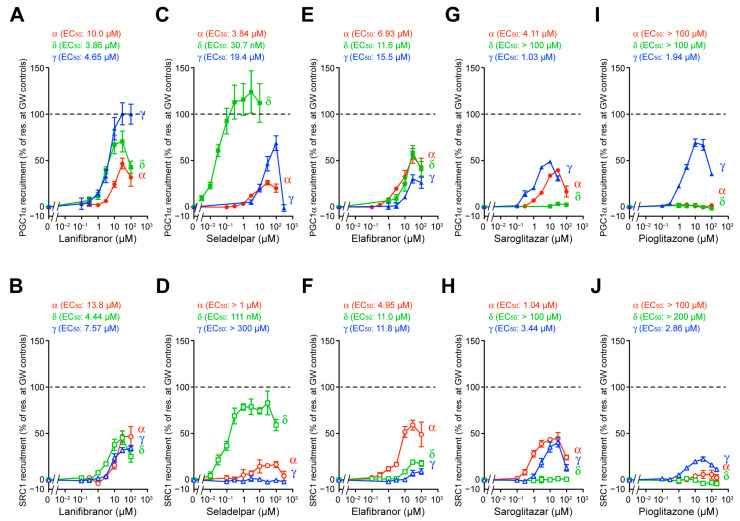
TR-FRET-based LBD-mediated human PPARα/δ/γ coactivator recruitment assay. The LBD-mediated PPARα/δ/γ recruitment of coactivator peptides, PGC1α and SRC1, was induced by PPARα-selective GW7647, PPARδ-selective GW501516, and PPARγ-selective GW1929 in a concentration-dependent manner, and their maximal responses (all at 1 µM) were considered as the 100% responses (dashed lines). The PGC1α (**A**,**C**,**E**,**G**,**I**) or the SRC1 (**B**,**D**,**F**,**H**,**J**) recruitment activities by lanifibranor (**A**,**B**), seladelpar (**C**,**D**), elafibranor (**E**,**F**), saroglitazar (**G**,**H**), and pioglitazone (**I**,**J**) were investigated. Data are presented as the mean ± SE of three or four independent experiments with duplicate samples, and the calculated EC_50_ values are shown.

The ligand-bound activated forms of PPARs are stabilized by associations with specific coactivators, thereby enabling the regulation of specific gene expression [24,26]. The different coactivator recruitment profiles via PPARγ-LBD were observed for rosiglitazone and lanifibranor [31]. The TR-FRET-based detection of the physical association between PPARα/δ/γ-LBD and their coactivators is a highly sensitive cell-free assay for evaluating the activities of PPAR ligands [14]. Again, the maximal effects exerted by the PPAR-selective full agonists (1 µM GW7647, GW501516, and GW1929) [14] for two types of coactivator peptides (PGC1α and SRC1) that have altered functions in terms of energy expenditure [25] were considered as the 100% recruitment responses [14]. Lanifibranor recruited both PGC1α and SRC1 to all PPARα/δ/γ-LBD structures with equivalent potencies but altered efficacies: γ > δ > α in the case of PGC1α (Figure 2A) and α ~ δ ~ γ in the case of SRC1 (Figure 2B). Seladelpar recruited both PGC1α and SRC1 to PPARδ-LBD with the highest potencies (30.7 nM and 111 nM, respectively) and efficacies (124% and 83.0%, respectively) compared to those of their recruitment to PPARα/γ-LBD (Figure 2C,D). It should be noted that seladelpar recruited PGC1α but not SRC1 to PPARγ-LBD (Figure 2C,D). Although elafibranor recruited PGC1α to all PPARα/δ/γ-LBD structures with similar potencies and efficacies (Figure 2E), it recruited SRC1 with a higher potency and efficacy to PPARα-LBD (4.95 µM and 59.2%) than to PPARδ/γ-LBD (Figure 2F). Saroglitazar recruited both PGC1α and SRC1 to PPARα/γ-LBD, but not to PPARδ-LBD (Figure 2G,H), while pioglitazone recruited both PGC1α and SRC1 to PPARγ-LBD, but not to PPARα/δ-LBD (only slightly to PPARα-LBD in the case of SRC1) (Figure 2I,J). Those recruitment profiles were found to be almost identical to their transactivation profiles, although each PPAR ligand displayed different activities toward PGC1α and SRC1.

### 3.3. Thermal Stability of PPARα/δ/γ-LBD

**Figure 3:**
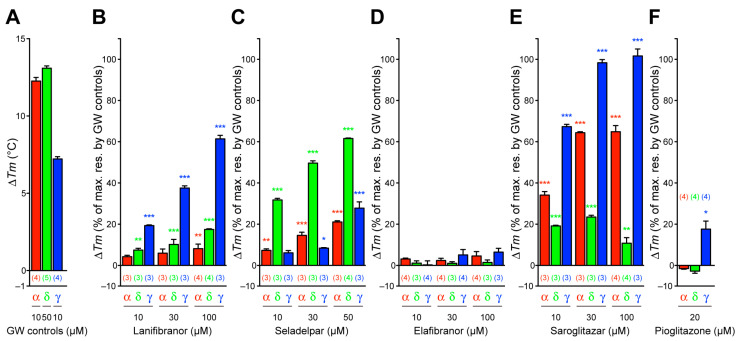
Circular dichroism-based PPARα/δ/γ-LBD thermal stability assay. The PPARα/δ/γ-LBD exhibited increased *T*_m_ values (∆*T*_m_) at 222 nm after an exposure to PPARα-selective GW7647, PPARδ-selective GW501516, and PPARγ-selective GW1929 at their highest soluble concentrations (10, 50, and 10 µM, respectively) (**A**) that were considered as the maximal (100%) responses. Relative ∆*T*_m_ responses by lanifibranor (**B**), seladelpar (**C**), elafibranor (**D**), saroglitazar (**E**), and pioglitazone (**F**) were measured as the reflection of the thermal stability of the α-helical structures in the PPARα/δ/γ-LBD. Data are presented as the mean ± SE of three or four (in parentheses) independent experiments. Differences vs. basal levels (with 0.1% DMSO) were statistically assessed by one-way ANOVA, followed by a Dunnett’s post hoc test: * *p* < 0.05, ** *p* < 0.01, and *** *p* < 0.001.

Nuclear receptors, including PPARs, display an increased thermal stability upon ligand binding, which is detectable through CD spectroscopy [32]. Ligand-induced alterations in the *T*_m_ values at 222 nm are considered as the reflection of stable α-helical structures in PPARs, because ligand binding stabilizes the ligand-binding portion (LBP) [33]. The basal (only solvent; 0.1% DMSO) *T*_m_ values were 49.54 °C ± 0.12 °C (*n* = 4), 51.76 °C ± 0.17 °C (*n* = 6), and 48.95 °C ± 0.16 °C (*n* = 4) for PPARα/δ/γ-LBD, respectively. The PPARα/δ/γ-selective GW compounds (GW7647, GW501516, and GW1929, respectively) indeed exhibited highly increased *T*_m_ values with their highest soluble (in 0.1% DMSO) concentrations (10, 50, and 10 µM, respectively) so as to match the 10 µM PPARα/δ/γ-LBD protein in the assay mixture (Figure 3A). Lanifibranor increased the *T*_m_ values in all PPARα/δ/γ-LBD complexes, with the highest values being evident in PPARγ-LBD (61.4%) (Figure 3A,B), which is similar to its effect upon the PGC1α recruitment (Figure 2A). Seladelpar also increased the *T*_m_ values in all PPARα/δ/γ-LBD complexes with the highest values being evident in PPARδ-LBD (Figure 3C), which is similar to all other assays (Figure 1C and Figure 2C,D). In contrast, elafibranor did not significantly alter the *T*_m_ values in the PPARα/δ/γ-LBD complexes (Figure 3D). Saroglitazar highly increased the *T*_m_ values in all PPARα/δ/γ-LBD complexes, with the highest values being evident in PPARγ-LBD and then in PPARα-LBD (Figure 3E), whereas pioglitazone increased the *T*_m_ values only slightly in the case of PPARγ-LBD and exerted no effects upon PPARα/δ-LBD (Figure 3F). The thermal stability experiments illustrated other facets of the PPAR activation by those ligands.

### 3.4. Structures of the PPARα/δ/γ-LBD–Lanifibranor Complexes

**Figure 4:**
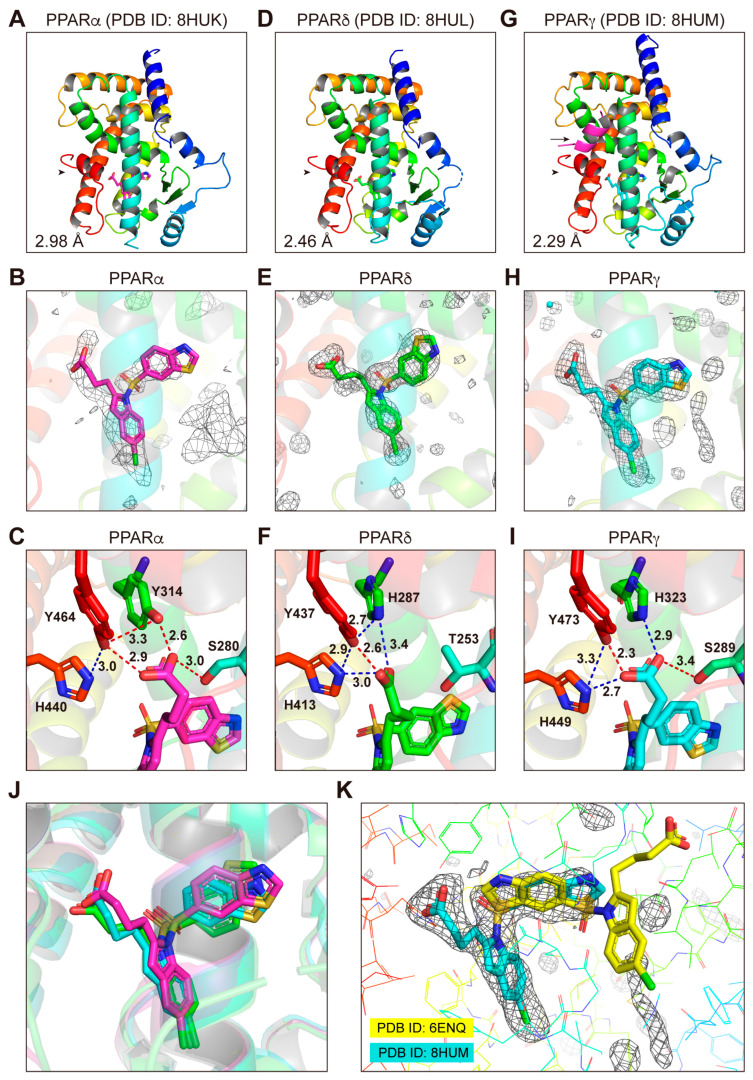
PPARα/δ/γ-LBD–lanifibranor cocrystal structures. Cocrystals of lanifibranor and PPARα-LBD (**A**–**C**), PPARδ-LBD (**D**–**F**), or PPARγ-LBD (**G**–**I**) were analyzed using X-ray diffraction. (**A**,**D**,**G**) Overall structures of the complexes deposited in PDB with IDs: 8HUK, 8HUL, and 8HUM, respectively. The SRC1 peptide (α-helix in magenta) and the AF-2 helix 12 (α-helix in red) are indicated by arrows (only in (**G**)) and arrowheads, respectively. The highest resolutions are labeled. (**B**,**E**,**H**) Magnified views of lanifibranor located in the “Center” region of PPARα/δ/γ-LBD. The electron density is shown in the mesh via *F*_o_–*F*_c_ omit maps contoured at +3.0 σ. A water molecule is presented as a cyan sphere in (**H**). (**C**,**F**,**I**) Hydrogen bonds and electrostatic interactions between lanifibranor and the four consensus amino acid residues (that recognize the carboxyl moiety of lanifibranor) are indicated by red and blue dotted lines, respectively, along with their distances (in Å). (**J**) Superposed view of lanifibranor in PPARα (magenta)/PPARδ (green)/PPARγ (cyan)-LBD cocrystal structures. (**K**) Superposed view of our *F*_o_–*F*_c_ omit maps of PPARγ-LBD–lanifibranor (the same with (**H**)) and lanifibranor in a previous PDB submission (ID: 6ENQ).

After some trial and error using various cocrystallization techniques that we applied for PPARα-LBD and its numerous ligands [15], including conventional cocrystallization with multiple buffer sets, cross-seeding, soaking, coactivator addition, and delipidation in order to remove endogenous fatty acids [16], the PPARα-LBD–lanifibranor cocrystals were obtained by soaking the PPARα-LBD–clofibric acid–SRC1 cocrystals [16] in a buffer containing lanifibranor (Figure S1A). X-ray diffraction analyses revealed their dimeric structures (PDB ID: 8HUK): one holding a single lanifibranor molecule between H3 and H7, but not the SRC1 peptide (Figure 4A,B), and another with SRC1, but not with lanifibranor (Figure S2A,B). The dimeric structure of the PPARδ-LBD–lanifibranor cocrystals (Figure S1B) was obtained using cocrystallization without coactivators (PDB ID: 8HUL), as both have a single lanifibranor molecule in the same position (Figure 4D,E and Figure S2C,D). The monomeric structure of PPARγ-LBD–lanifibranor cocrystals (Figure S1C) was obtained using cocrystallization with SRC1 (PDB ID: 8HUM) (Figure 4G,H). The structures of lanifibranor bound to PPARα/δ/γ-LBD were solved in a monoclinic space group *P*2_1_ at a 2.98 Å resolution, a monoclinic space group *P*2_1_ at a 2.46 Å resolution, and an orthorhombic space group *P*2_1_2_1_2_1_ at a 2.29 Å resolution (Table S1). Obtained structures were identical to previously reported active conformations [14,15,17] that form the Activation Function-2 (AF-2) helix 12, thereby providing root mean square distances of 0.60 Å (226 common Cα positions in PPARα/δ) and 0.53 Å (212 common Cα positions in PPARα/γ) (Figure 4A,D,G).

The carboxylic groups of the PPAR ligands are known to stabilize the AF-2 helix 12 through hydrogen bonds (red dotted lines) and electrostatic interactions (blue dotted lines) with the four surrounding consensus amino acids [14]: Ser280/Tyr314/His440/Tyr464 in PPARα-LBD (Figure 4C), Thr253/His287/His413/Tyr437 in PPARδ-LBD (Figure 4F), and Ser289/His323/His449/Tyr473 in PPARγ-LBD (Figure 4I). However, a very close (2.3-Å) proximity was observed in the case of Y473 of PPARγ and lanifibranor (Figure 4I). We have previously defined five regions in PPARα-LBP (Arms I–III/X and Center) [15] and four regions (Arms II/III/X and Center) in PPARδ/γ-LBP [14]. Lanifibranor was found to locate in the same position of the Center region in PPARα/δ/γ-LBD, although its benzothiazole moiety in PPARδ was flipped sideways when compared to that in PPARα/γ (Figure 4J). A previous cocrystallization study [31] located lanifibranor in the “Center” (for its benzothiazole ring), the “Arm II” (for its 5-chloroindole moiety), and the “Arm III” (for its carboxylic moiety) regions (yellow in Figure 4K); however, in this study, no electron density was observed in the “Arm III” region (Figure 4K). Our structure seems more reasonable because the interaction of the carboxylic groups of lanifibranor with the four consensus amino acids contributes to the stabilization of the AF-2 helix 12 so as to facilitate the recruitment of coactivators in all PPAR subtypes.

### 3.5. Structures of the PPARα/δ/γ-LBD–Seladelpar Complexes

**Figure 5:**
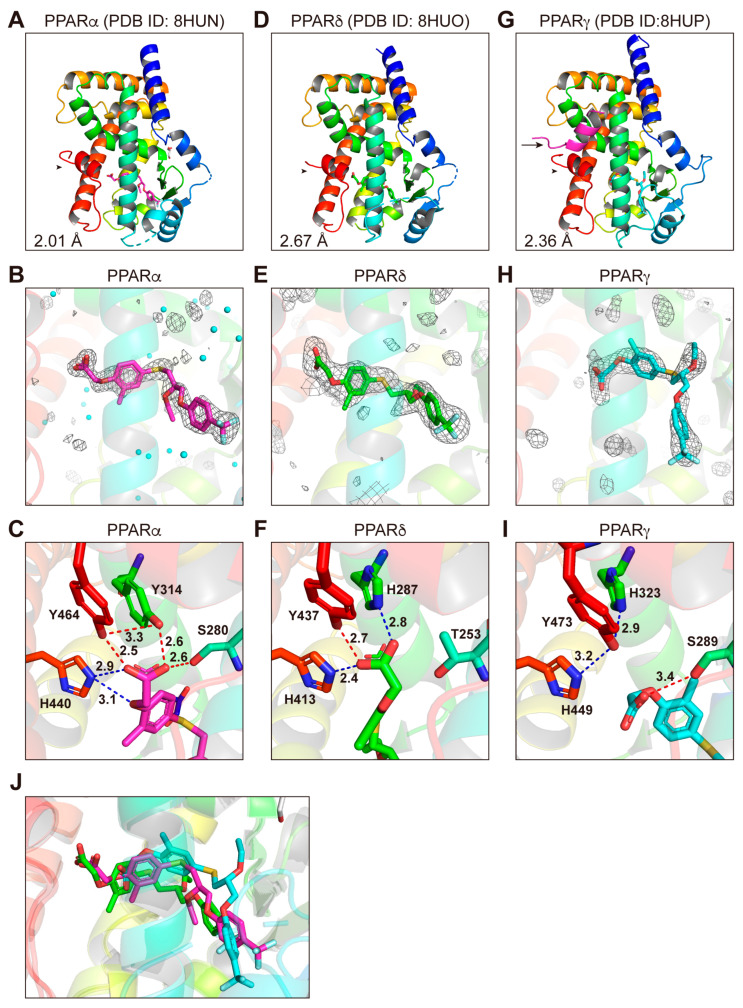
PPARα/δ/γ-LBD–seladelpar cocrystal structures. Cocrystals of seladelpar and PPARα-LBD (**A**–**C**), PPARδ-LBD (**D**–**F**), or PPARγ-LBD (**G**–**I**) were analyzed using X-ray diffraction. (**A**,**D**,**G**) Overall structures of the complexes deposited in PDB with IDs 8HUN, 8HUO, and 8HUP, respectively. The SRC1 peptide (α-helix in magenta) and the AF-2 helix 12 (α-helix in red) are indicated by arrows (only in (**G**)) and arrowheads, respectively. The highest resolutions are labeled. (**B**,**E**,**H**) Magnified views of seladelpar located in the “Center” and the “Arm II” regions of PPARα/δ/γ-LBD. The electron density is shown in the mesh via *F*_o_–*F*_c_ omit maps contoured at +3.0 σ. Water molecules are presented as cyan spheres in (**B**). (**C**,**F**,**I**) Hydrogen bonds and electrostatic interactions between seladelpar and the four consensus amino acid residues (that recognize the carboxyl moiety of seladelpar) are indicated by red and blue dotted lines, respectively, along with their distances (in Å). (**J**) Superposed view of seladelpar in PPARα (magenta)/PPARδ (green)/PPARγ (cyan)-LBD cocrystal structures.

Due to the fact that seladelpar is a relatively low-affinity PPARα/γ ligand (Figure 1C, Figure 2C,D and Figure 3C), its cocrystals were obtained by cocrystallization with a delipidized PPARα-LBD [15] or with PPARγ-LBD and SRC1 (Figure S1D,F). In contrast, the PPARδ-LBD–seladelpar cocrystals were obtained by employing cocrystallization without coactivators (Figure S1E). X-ray diffraction has revealed the monomeric structures for PPARα (Figure 5A,B) and PPARγ (Figure 5G,H), as well as the dimeric structure for PPARδ (Figure 5D,E and Figure S2E,F). The structures of seladelpar bound to PPARα/δ/γ-LBD were solved in a monoclinic space group *P*2_1_ at a 2.01 Å resolution (PDB ID: 8HUN), a monoclinic space group *P*2_1_ at a 2.67 Å resolution (PDB ID: 8HUO), and an orthorhombic space group *P*2_1_2_1_2_1_ at a 2.36 Å resolution (PDB ID: 8HUP), respectively (Table S1). The electron density maps located a single seladelpar molecule per PPARα/δ/γ-LBD protein monomer (Figure 5B,E,H, and Figure S2F). Seladelpar was located in the similar positions of the asymmetric units (Figure 5B and Figure S2F). The obtained PPARα/δ/γ-LBD helical structures were identical to those of previously reported active conformations described for lanifibranor above. Seladelpar was found to be located in the similar position of the “Center” and the “Arm II” regions in PPARα/δ/γ-LBD; however, its orientations differed (Figure 5J). Likewise, the orientations of the carboxylic group of seladelpar toward the four consensus amino acids in PPARα/δ/γ-LBD (Figure 5C,F,I) were different from those of lanifibranor (Figure 4C,F,I). The loss of the interaction with His323/His449/Tyr473 in PPARγ (Figure 5I) might explain the weak activity of seladelpar against PPARγ.

### 3.6. Structures of the PPARα-LBD–Elafibranor Complex

**Figure 6:**
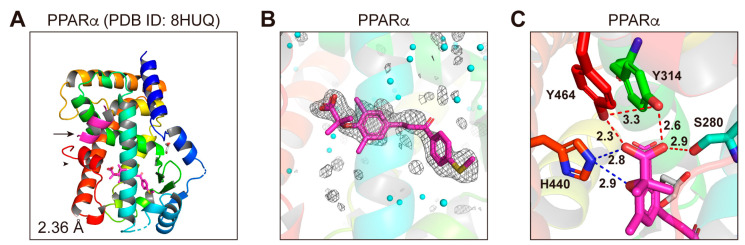
PPARα-LBD–elafibranor cocrystal structures. A cocrystal of elafibranor and PPARα-LBD (**A**–**C**) was analyzed using X-ray diffraction. (**A**) The 2.36 Å resolution overall structure of the complex deposited in PDB ID: 8HUQ. The SRC1 peptide (α-helix in magenta) and the AF-2 helix 12 (α-helix in red) are indicated by an arrow and an arrowhead, respectively. (**B**) Magnified view of elafibranor located in the “Center” and the “Arm II” regions of PPARα-LBD. The electron density is shown in the mesh via *F*_o_-*F*_c_ omit maps contoured at +3.0 σ. Water molecules are presented as cyan spheres. (**C**) Hydrogen bonds and electrostatic interactions between elafibranor and the four consensus amino acid residues (that recognize the carboxyl moiety of elafibranor) are indicated by red and blue dotted lines, respectively, along with their distances (in Å).

The PPARα-LBD–elafibranor cocrystals were obtained by soaking the PPARα-LBD–clofibric acid–SRC1 cocrystals [15] in a buffer containing elafibranor (Figure S1G). X-ray diffraction analyses revealed the monomeric structure with a single elafibranor and SRC1 (Figure 6A,B). The complex structure was solved in a monoclinic space group *P*2_1_ at a 1.65 Å resolution (Table S1; PDB ID: 8HUQ). The obtained structures were identical to the previously reported active conformations described above, while hydrogen bond/electrostatic interactions were observed between the carboxylic group of elafibranor and Ser280/Tyr314/His440/Tyr464 in PPARα-LBD (Figure 6C). Regrettably, we failed to obtain PPARδ/γ-LBD–elafibranor cocrystals (Figure S1H,I), although elafibranor did activate PPARδ/γ to some extent (Figure 1D and Figure 2E,F).

### 3.7. LBP Regional Localization of the Five PPAR Ligands in PPARα/δ/γ-LBD

**Figure 7:**
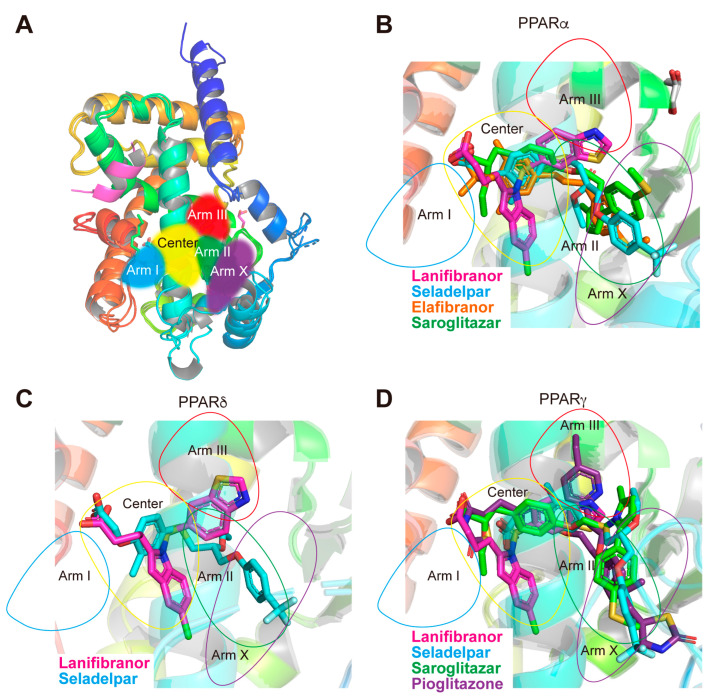
Ligand-binding pocket (LBP) regional localization of five PPAR ligands in PPARα/δ/γ-LBD. (**A**) LBP comprising the “Center” and the “Arms I–III and X” regions of PPARα/δ/γ-LBD [15]. (**B**) Superimposed image of lanifibranor (magenta; from 8HUK), seladelpar (cyan; 8HUN), elafibranor (orange; 8HUQ), and saroglitazar (green; 6LXC) [15] in PPARα-LBD. (**C**) Superimposed image of lanifibranor (magenta; 8HUL) and seladelpar (cyan; 8HUO) in PPARδ-LBD. (**D**) Superimposed image of lanifibranor (magenta; 8HUM), seladelpar (cyan; 8HUP), saroglitazar (green; 7E0A) [17], and pioglitazone (purple; 2XKW) in PPARγ-LBD.

PPARα/δ/γ-LBP comprises five regions (Figure 7A) [14,15,17]. In PPARα-LBP, seladelpar, elafibranor, and saroglitazar (PDB ID: 6LXB/6LXC using different crystallization methods) [15] were located in the “Center” and in the “Arm II/X” regions, whereas lanifibranor was only found in the “Center” (Figure 7B). In PPARδ-LBP, lanifibranor was located in the “Center,” and seladelpar was located in the “Center” and the “Arm II” regions (Figure 7C). In PPARγ-LBP, lanifibranor was only found in the “Center” while seladelpar and saroglitazar (PDB ID: 7E0A) [17] were found in the “Center” and the “Arm II/X” regions. Mueller et al. deposited a PPARγ-LBD–pioglitazone structure in the PDB, in which two pioglitazone molecules were located in LBP (PDB ID: 2XKW; not published in a paper): one was in the “Center” and the “Arms II/X” regions and the other was in the “Arms II/III” (Figure 7D). These results indicate that each PPAR ligand has a flexible molecular frame allowing it to bind to different regions of the PPARα/δ/γ-LBPs.

### 3.8. Molecular Interactions between PPARα/δ/γ-LBD Amino Acids and the Ligands

For more detailed comparisons of ligand binding loci, proximity distances (≤4.5 Å) between each amino acid in PPARα/δ/γ-LBD and the ligands (lanifibranor, seladelpar, and elafibranor) were measured using PyMOL, and the MolDock scores (a kind of binding potential) of their interactions were calculated (Table S2). The four consensus amino acids surrounding the carboxylic groups of the ligands were stabilized by ligand binding (as manifested by minus MolDock scores), except for PPARγ-LBD–seladelpar, in which hydrogen bonds were not formed between His323/Tyr473 and the ligand (Figure 5I). Some other consensus amino acids, such as Ile272/Cys276/Phe318/Leu321 in PPARα-LBD, Val245/Cys249/Phe291/Leu294 in PPARδ-LBD, and Ile281/Cys285/Tyr327/Leu330 in PPARγ-LBD, were stabilized by all three ligands, whereas some consensus amino acids (i.e., Leu247/Ala250/Val255/Ala333 in PPARα-LBD and Leu255/Glu259/Phe264/Ser342 in PPARγ-LBD) were specifically stabilized by seladelpar and other consensus amino acids (i.e., Leu347/Phe351, Leu320/Phe324 and Leu356/Phe360 in PPARα/δ/γ-LBD, respectively) were stabilized only by lanifibranor (Table S2).

## 4. Discussion

This study investigated how lanifibranor, seladelpar, and elafibranor bind to and activate PPARα/δ/γ subtypes. Major antioxidant genes, including catalase, heme oxygenase-1, glutathione peroxidase 3/4, superoxide dismutase 2/3, thioredoxin, CD36, and uncoupling protein 2/3, contain PPAR responsive element (PPRE) in their promoter regions and are transcriptionally regulated by the PPARs [34,35,36]. In addition, a putative PPRE is present in the promoter region of Nrf2 that is one of the most important regulators of cellular responses to oxidative stress and inflammation [37]. Therefore, such PPAR agonists might potently modulate systemic redox homeostasis and inflammation as well as lipid/insulin signaling to counteract NASH.

Lanifibranor, the PPARα/δ/γ pan agonist developed by Inventiva (Daix, France) [31], was first described for its prevention of experimental skin [38] and lung fibrosis [39], and was then applied to liver fibrosis [40]. In a phase 2b study involving 247 patients with active NASH (NCT03008070), lanifibranor significantly improved general NASH conditions [41]. A large-scale (~1000 patients) phase 3 study, evaluating the long-term efficacy and safety of lanifibranor in adult NASH patients with Fibrosis 2/3 stage of liver fibrosis (NCT04849728) is currently underway, and its results have not been posted yet. The EC_50_ values for PPARα/δ/γ were in small ranges (0.4–5 µM in Figure 1B and 3–14 µM in Figure 2A,B), and therefore, lanifibranor in clinical doses seems to be able to activate all PPAR subtypes at once. Due to the fact that its efficacies for PPARγ activation often match with those of pioglitazone (Figure 2A vs. Figure 2I, Figure 2B vs. Figure 2J, and Figure 3B vs. Figure 3F), PPARγ-related undesirable side effects (such as weight gain, edema, bone loss, and congestive heart failure) [42] should be closely monitored in the case of lanifibranor. In the cocrystal structures, indeglitazar, another PPARα/δ/γ pan agonist that resembles lanifibranor in structure, has also been reported to exist in the similar “Center” regions in PPARα/δ/γ (PDB ID: 3ET1, 3ET2, and 3ET3, respectively) [43] and some consensus amino acids in the Center region (such as Phe273/Ile354, Phe246/Ile327, and Phe282/Phe363 in PPARα/δ/γ-LBD, respectively) were highly stabilized by lanifibranor (Table S2); therefore, such molecular frames might be favorable for a PPAR pan activity.

Seladelpar is the novel PPARδ-selective agonist developed by CymaBay Therapeutics (Newark, NJ, USA) [44]. Although its clinical trial against NAFLD/NASH was once discontinued at phase 2 due to abnormal findings on liver biopsy [9], the FDA thereafter lifted the injunction on July 2020 due to subsequent doubts about the relevance of the drug [45]. The clinical trials have not resumed since then, and thus, its therapeutic potential against NASH remains unknown. In its clinical trial against hyperlipidemia (NCT00701883), seladelpar reduced the assessed blood lipid parameters (triglyceride, total cholesterol, LDL-cholesterol, and free fatty acid levels), the alkaline phosphatase and γ-glutamyl transferase (GGT) activities, and the homeostatic model assessment–insulin resistance [46]. Moreover, prompted by its positive results in its phase 2 clinical trial for primary biliary cholangitis, a phase 3 clinical trial is currently underway [44,47]. In mice, seladelpar has been reported to reverse dyslipidemia and the hepatic storage of lipotoxic lipids, thereby improving the NASH pathology in atherogenic-diet-fed obese diabetic mice [48]. Seladelpar could activate all PPAR subtypes; however, the EC_50_ values for PPARδ-LBD were ~2 orders lower than those for PPARα/γ-LBD (Figure 1C and Figure 2C,D), and this is why seladelpar can be used as a PPARδ-selective agonist in clinical use. In the cocrystal structures, seladelpar was bound to the “Center” and the “Arm II” regions of all PPARα/δ/γ-LBDs (Figure 5J), similar to the PPARδ-selective full agonist GW501516 in PPARδ-LBD (PDB ID: 5U46) [49]. Both carboxylic and trifluoromethyl groups of seladelpar were located in positions similar to those of GW501516, thereby implying that amino acid residues in those regions were important for the full activation of PPARδ. Indeed, Val298, Leu303, Val312, and Ile328, which were reported to be important for the binding to GW501516 and other synthetic PPARδ-selective ligands [49], were all stabilized by seladelpar (Table S2).

Elafibranor, the PPARα/δ dual agonist developed by Genfit (Loos, France), has been abandoned at the phase 3 clinical trial against NAFLD/NASH due to its non-significant effect on the primary endpoint of the resolution of NASH without a worsening of fibrosis [10]. In a phase 2 clinical trial (NCT01694849), its efficacy and safety at 80 and 120 mg/day doses for 52 weeks were confirmed among the 275 participating NASH patients [50]. Elafibranor also reduced the fasting plasma triglyceride levels and GGT activities, increased HDL cholesterol, and decreased insulin resistance and fasting plasma glucose levels, in abdominally obese patients with either combined dyslipidemia or prediabetes [51]. Further clinical trials have suggested that elafibranor can improve peripheral and hepatic insulin sensitivity [52]. As its EC_50_ values for PPARα/δ/γ-LBD were in small ranges (0.4–3 µM in Figure 1D and 5–16 µM in Figure 2E,F), elafibranor in clinical doses seems to be able to activate all PPAR subtypes at once. The efficacy of elafibranor for PPARγ activation was lower than that of pioglitazone in the cases of transactivation (Figure 1D,F), PGC1α (Figure 2E,I), and SRC1 recruitment (Figure 2F,J). Interestingly, the impact of elafibranor on the thermal stability of PPARα/δ/γ-LBD was much weaker than that of other ligands (Figure 3D). Regrettably, we failed to obtain PPARδ/γ-LBD–elafibranor cocrystals (Figure S1H,I). Therefore, it might be difficult for elafibranor to form stable complexes with any of the PPAR subtypes, especially with PPARδ/γ, and to induce enough clinical impact in some cases (e.g., NASH).

When compared to the LBPs of other nuclear receptors that have 600–1100 Å^3^ cavities, the LBPs of PPARα/δ/γ have relatively large (1300–1440 Å^3^) cavities that are able to accept 1–4 ligand molecules [15]. The PPARα/δ/γ-LBD–various ligands’ cocrystal structures registered in the PDB until 18 July 2023 (60, 52, and 288, respectively; among which 40, 8, and 25 registrations, respectively, derive from our laboratories) demonstrate their extremely diverse ligand binding modes. We have, herein, added seven novel structures (three, two, and two for PPARα/δ/γ, respectively) with altered ligand locations. However, unfortunately, we failed to provide a clear molecular basis (such as close interactions with specific PPAR-LBD amino acids; Table S2) for PPARα/δ/γ targeting preferences of these three drugs, perhaps in part for large PPAR-LBPs, which is the limitation of this study. Nevertheless, future drug discovery for NASH will undoubtedly benefit from functional and structural investigation of the PPARα/δ/γ–ligand molecular interactions such as in this study. Lanifibranor (rather than elafibranor) could be a prototype of PPAR pan agonists that bind to the similar positions in PPARα/δ/γ-LBD and thus have comparable preferences for PPARα/δ/γ, and seladelpar could be a lead compound of upcoming PPARδ-selective agonists with more preferences to PPARδ and less preferences to PPARα/γ.

## Data Availability

The X-ray diffraction datasets have been deposited in the PDB with accession numbers: 8HUK, 8HUL, 8HUM, 8HUN, 8HUO, 8HUP, and 8HUQ.

## Supplementary Materials

The following supporting information can be downloaded at: https://www.mdpi.com/article/10.3390/antiox12081523/s1, Figure S1: Snapshots of the crystals as mounted on the BL5A or BL17A beamline goniometers; Figure S2: Other subunit structures of PPARα/δ dimers in the cocrystals; Table S1: Data collection and refinement statistics (molecular replacement); Table S2 (A separate Excel file): All PPARα/δ/γ-LBD amino acids that have ≤4.5 Å proximity distances from lanifibranor, seladelpar, and elafibranor, and the MolDock scores of their molecular interactions.
