# Supplementary material for: Functional and Structural Insights into the Human PPARα/δ/γ Targeting Preferences of Anti-NASH Investigational Drugs, Lanifibranor, Seladelpar, and Elafibranor
Source: Antioxidants (Basel). 2023 Jul 29;12(8):1523. doi: 10.3390/antiox12081523 (PMC10451623; doi:10.3390/antiox12081523)
Supplement: Supplementary file 1 [file antioxidants-12-01523-s001.zip › Supproting Information.pdf]

Supporting Information

Functional and structural insights into the human PPARα/δ/γ targeting preferences of anti-NASH investigational drugs, lanifibranor, seladelpar, and elafibranor

Shotaro Kamata[1], Akihiro Honda[1], Ryo Ishikawa[1], Makoto Akahane[1], Ayane Fujita[1], Chihiro Kaneko[1], Saeka Miyawaki[1], Yuki Habu[1], Yui Shiiyama[1], Kie Uchii[1], Yui Machida[1], Takuji Oyama[2], and Isao Ishii[1,*]

[1]Department of Health Chemistry, Showa Pharmaceutical University, Machida, Tokyo 194-8543, Japan

[2]Faculty of Life and Environmental Sciences, University of Yamanashi, Kofu, Yamanashi 400-8510, Japan

*Correspondence

Contents:

Figure S1

Snapshots of the crystals as mounted on the BL5A or BL17A beamline goniometers

Figure S2

Other subunit structures of PPARα/δ dimers in the cocrystals

Table S1

Data collection and refinement statistics (molecular replacement)

Table S2 (A separate Excel file)

All PPARα/δ/γ-LBD amino acids that have ≤4.5 Å proximity distances from lanifibranor, seladelpar, and elafibranor, and the MolDock scores of their molecular interactions

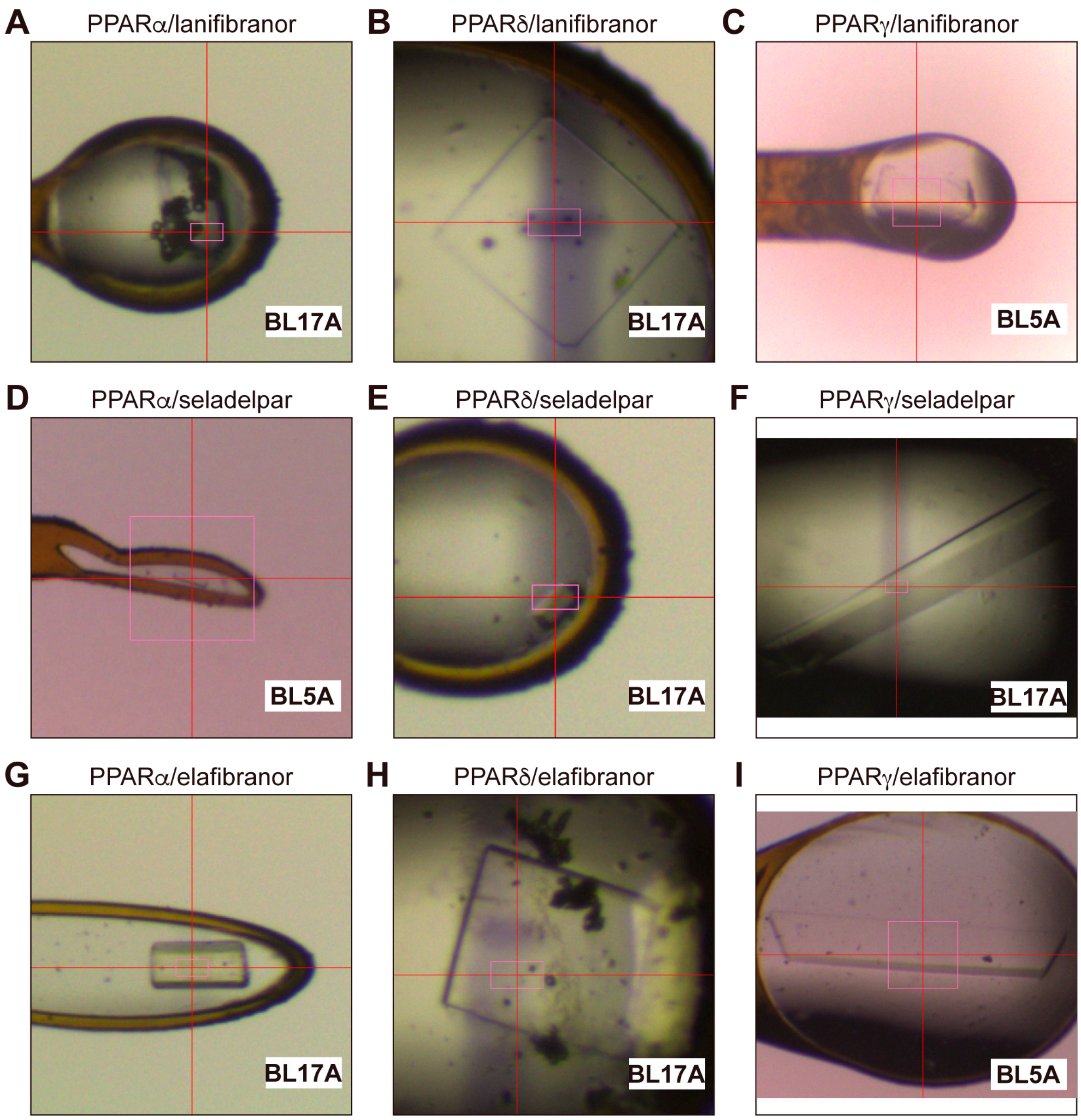

Figure S1. Snapshots of the crystals as mounted on the BL5A or BL17A beamline goniometers. Cocrystals of PPARα/δ/γ-LBD–lanifibranor (**A**, **B**, and **C**, respectively), seladelpar (**D**, **E**, and **F**, respectively), and elafibranor (**G**, **H**, and **I**, respectively; cocrystals with PPARδ/γ-LBD were not obtained) were analyzed by X-ray diffraction. The sizes of the red square (in BL5A) and rectangular (in BL17A) meshes are 100×100 and 40×20 μm, respectively.

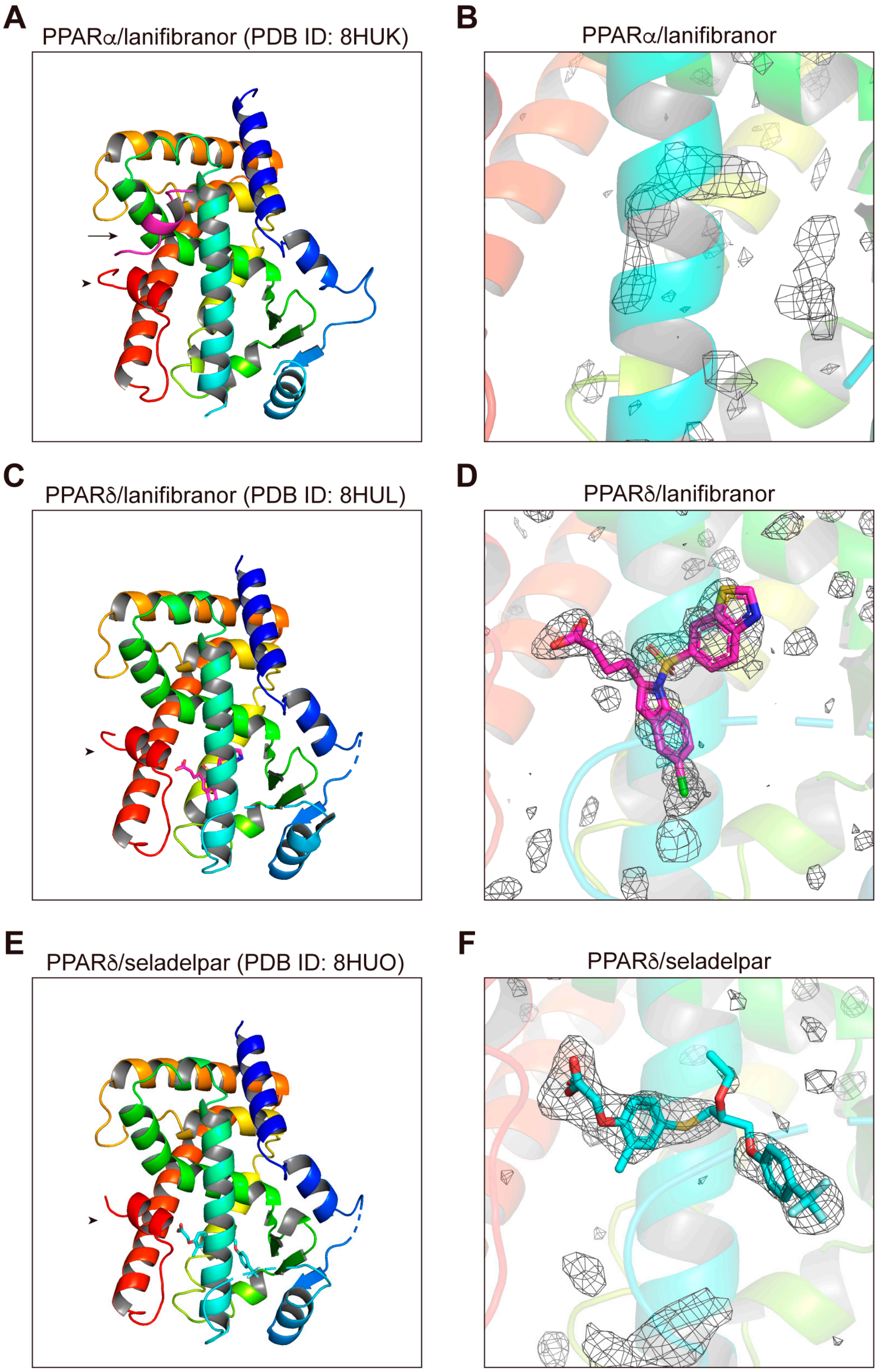
A
PPARα/lanifibranor (PDB ID: 8HUK)
B
PPARα/lanifibranor
C
PPARδ/lanifibranor (PDB ID: 8HUL)
D
PPARδ/lanifibranor
E
PPARδ/seladelpar (PDB ID: 8HUO)
F
PPARδ/seladelpar

Figure S2. Other subunit structures of PPARα/δ dimers in the cocrystals. Other subunits of the PPARα-LBD–lanifibranor cocrystals (**A** and **B**; 8HUK), the PPARδ-LBD–lanifibranor cocrystals (**C** and **D**; 8HUL), and the PPARδ-LBD–seladelpar cocrystals (**E** and **F**; 8HUO) were revealed by X-ray diffraction. (**A**, **C**, and **E**) The overall structures of the complexes. The SRC1 peptide (α-helix in magenta) and the AF-2 helix 12 (α-helix in red) are indicated by arrows and arrowheads, respectively. (**B**, **D**, and **F**) Magnified views of lanifibranor or seladelpar in other subunits of the PPARα/δ-LBD. The electron density is shown in the mesh via F_o-F_c omit maps contoured at +3.0σ.

Table S1. Data collection and refinement statistics (molecular replacement)

Protein	hPPARα-LBD	hPPARδ-LBD	hPPARγ-LBD
Peptide	SRC1	none	SRC1
Binding ligand	Lanifibranor	Lanifibranor	Lanifibranor
PDB ID	8HUK	8HUL	8HUM
**Data collection**			
Space group	$P2_1$	$P2_1$	$P2_12_12_1$
Cell dimensions			
a, b, c (Å)	59.73, 101.38, 60.36	39.17, 94.95, 96.93	49.86, 63.73, 123.48
α, β, γ (°)	90.00, 98.48, 90.00	90.00, 97.78, 90.00	90.00, 90.00, 90.00
Resolution (Å)	45.48–2.98 (3.16–2.98)	48.02–2.46 (2.56–2.46)	46.23–2.29 (2.37–2.29)
R_{merge}	0.050 (0.315)	0.067 (0.350)	0.041 (0.355)
R_{pim}	0.032 (0.211)	0.042 (0.217)	0.018 (0.150)
$CC_{1/2}$	0.999 (0.949)	0.997 (0.916)	0.999 (0.984)
$I / \sigma I$	12.3 (3.0)	12.3 (3.7)	21.6 (3.9)
Completeness (%)	99.3 (98.8)	98.9 (97.9)	99.8 (99.9)
Redundancy	3.4 (3.1)	3.5 (3.5)	6.4 (6.5)
**Refinement**			
Resolution (Å)	39.202–2.981	38.805–2.461	39.270–2.290
No. reflections	14,434	25,149	18,258
R_{work} / R_{free}	0.1974/0.2537	0.2109/0.2433	0.2057/0.2504
No. atoms			
Protein	4,194	4,155	2,327
Ligand	28	56	28
Water	0	0	4
B-factors	100.12	41.56	60.53
Protein	100.04	41.45	60.51
Ligand	111.12	49.19	64.08
Water			48.14
Ramachandran plot (%)			
Favored	93.63	96.41	92.93
Allowed	5.02	3.39	4.24
Outliers	1.35	0.20	2.83
R.m.s. deviations			
Bond lengths (Å)	0.001	0.002	0.008
Bond angles (°)	0.358	0.42	1.03

Values in parentheses are for highest-resolution shell.

Table S1. (Continued)

Protein	hPPARα-LBD	hPPARδ-LBD	hPPARγ-LBD
Peptide	none	none	SRC1
Binding ligand	Seladelpar	Seladelpar	Seladelpar
PDB ID	8HUN	8HUO	8HUP
**Data collection**			
Space group	$P2_1$	$P2_1$	$P2_12_12_1$
Cell dimensions			
a, b, c (Å)	44.57, 61.51, 53.18	39.47, 93.62, 96.67	49.81, 64.19, 123.92
α, β, γ (°)	90.00, 106.29, 90.00	90.00, 97.00, 90.00	90.00, 90.00, 90.00
Resolution (Å)	42.78–2.01 (2.06–2.01)	47.97–2.67 (2.80–2.67)	46.22–2.36 (2.45–2.36)
R_{merge}	0.078 (0.354)	0.044 (0.289)	0.037 (0.377)
R_{pim}	0.050 (0.229)	0.028 (0.179)	0.015 (0.154)
$CC_{1/2}$	0.997 (0.868)	0.999 (0.940)	1.000 (0.984)
$I / \sigma I$	11.3 (3.1)	18.2 (4.0)	28.0 (5.1)
Completeness (%)	99.8 (99.8)	99.5 (99.7)	99.9 (99.9)
Redundancy	3.4 (3.3)	3.5 (3.6)	6.6 (6.9)
**Refinement**			
Resolution (Å)	42.778–2.010	33.504–2.671	46.218–2.360
No. reflections	18,454	19,752	16,945
R_{work} / R_{free}	0.2001/0.2283	0.1946/0.2521	0.2049/0.2338
No. atoms			
Protein	2,150	4,155	2,331
Ligand	36	60	30
Water	69	0	0
B-factors	23.46	56.73	58.56
Protein	23.42	56.75	58.32
Ligand	29.21	55.75	77.69
Water	21.87		
Ramachandran plot (%)			
Favored	98.43	95.63	92.98
Allowed	1.57	3.78	4.91
Outliers	0.00	0.60	2.11
R.m.s. deviations			
Bond lengths (Å)	0.003	0.005	0.004
Bond angles (°)	0.50	0.72	0.75

Values in parentheses are for highest-resolution shell.

Table S1. (Continued)

Protein	hPPARα-LBD
Peptide	SRC1
Binding ligand	Elafibranor
PDB ID	8HUQ
**Data collection**	
Space group	$P2_1$
Cell dimensions	
a, b, c (Å)	44.76, 61.57, 53.35
α, β, γ (°)	90.00, 106.57, 90.00
Resolution (Å)	42.90–1.65 (1.68–1.65)
R_{merge}	0.022 (0.393)
R_{pim}	0.014 (0.240)
$CC_{1/2}$	1.000 (0.870)
I / σI	17.6 (3.2)
Completeness (%)	95.5 (95.5)
Redundancy	3.5 (3.6)
**Refinement**	
Resolution (Å)	32.799–1.650
No. reflections	31,930
R_{work} / R_{free}	0.1864/0.2135
No. atoms	
Protein	2,165
Ligand	33
Water	85
B-factors	34.10
Protein	33.89
Ligand	46.38
Water	34.69
Ramachandran plot (%)	
Favored	97.67
Allowed	2.33
Outliers	0.00
R.m.s. deviations	
Bond lengths (Å)	0.013
Bond angles (°)	1.26

Values in parentheses are for highest-resolution shell.
